# Supplementary material for: Ultra-compact snapshot spectral light-field imaging
Source: Nat Commun. 2022 May 18;13:2732. doi: 10.1038/s41467-022-30439-9 (PMC9117667; doi:10.1038/s41467-022-30439-9)
Supplement: Supplementary file 1 — Supplementary Information [file 41467_2022_30439_MOESM1_ESM.docx]

Supplementary information for

Ultra-compact snapshot spectral light-field imaging

Xia Hua^1,†^, Yujie Wang^2,†^, Shuming Wang^1,3,4,†,*^, Xiujuan Zou^1^, You Zhou^1^, Lin Li^5,6^, Feng Yan^1^, Xun Cao^1,3,4*^, Shumin Xiao^2,*^, Din Ping Tsai^5,*^, Jiecai Han^7^, Zhenlin Wang^1,4,*^, and Shining Zhu^1,3,4,*^

^1^National Laboratory of Solid State Microstructures, School of Physics, School of Electronic Science and Engineering, Nanjing University, Nanjing, 210093, China

^2^Ministry of Industry and Information Technology Key Lab of Micro-Nano Optoelectronic Information System, Harbin Institute of Technology (Shenzhen), Shenzhen, 518055, China

^3^Key Laboratory of Intelligent Optical Sensing and Manipulation, Ministry of Education, Nanjing University, Nanjing, 210093, China

^4^Collaborative Innovation Center of Advanced Microstructures, Nanjing, 210093, China

^5^Department of Electrical Engineering, City University of Hong Kong, Kowloon, Hong Kong, China

^6^State Key Laboratory of Precision Spectroscopy, School of Physics and Electronic Science, East China Normal University, Shanghai, 200062, China

^7^National Key Laboratory of Science and Technology on Advanced Composites in Special Environments, Harbin Institute of Technology, Harbin 150080, China.

^†^These authors contributed equally: Xia Hua, Yujie Wang, Shuming Wang.

Emails: wangshuming@nju.edu.cn, caoxun@nju.edu.cn, shumin.xiao@hit.edu.cn, dptsai@cityu.edu.hk, zlwang@nju.edu.cn, zhusn@nju.edu.cn.

**Supplementary Note 1: Design and numerical simulation**

All numerical simulations are carried out using commercial software based on the CST Studio Suite. TiO_2_ nanopillars and the inverse structures are chosen as the building blocks with high refractive-index and low loss. For the structural optimization of TiO_2_ nanostructures, the size parameters of unit cells are determined by evaluating their transmission spectra and the phase shifts. The perfectly matched layer and periodic boundary condition are plotted as the x- and y-directions, respectively, to estimate the focal length of the designed metalens. Supplementary Fig. 1 shows the schematic picture of the unit cells, and their detail size parameters are summarized in Supplementary Table. 1. The corresponding phase and efficiency information are plotted in Supplementary Fig. 2. The refractive index of TiO_2_ is obtained from experimental data inSupplementary Fig. 3.

**Supplementary Note 2: Device fabrication**

Film deposition: The high quality TiO_2_ film is deposited onto a 13 nm indium tin oxide (ITO) coated glass substrate with electro-beam evaporator (Syskey A-75) with deposition rate of 0.8 Å/s and base vacuum pressure of 2×10^-7^ torr. The optical parameters are measured using spectroscopic ellipsometry and shown in Supplementary Fig. 3. The real part of refractive index is above 2.1 through the whole visible wavelength while the loss is almost ignorable.

Nanofabrication: After the deposition of TiO_2_, 120 nm PMMA A2 is spin-coated onto the TiO_2_ film and baked at 180℃ for 10 minutes. Then the PMMA film is exposed to electro-beam with an electron-beam lithography system (Raith E-line Plus). After developed in MIBK: IPA solution at 20℃, the designed nanostructures are patterned in the PMMA film. Then the sample is transferred into an E-beam evaporator and 40 nm chromium (Cr) is deposited onto it. Finally, the pattern is transferred to Cr through lift off process using Remover PG.

By applying the Cr as the hard make, the TiO_2_ film is etched with Cl_2_ and Ar mixed gas in an inductively coupled plasma etcher (Oxford ICP100). The selectivity between Cr and TiO_2_ is about 50:1 and the etching speed for TiO_2_ is about 20 nm/s. The final TiO_2_ metalens is achieved by removing the Cr mask in chromium etchant at room temperature for 2 minutes, as shown in Supplementary Fig. 4.

**Supplementary Note 3: Optical measurements**

**3.1 The efficiency of single metalens**

The focusing performances of the metalens are characterized using a custom-built optical system shown in Supplementary Fig. 5. A white LED lamp is used as the broadband light source to illuminate the target object. Circularly polarized light is generated by linear polarizer and a quarter-wave plate. An objective (5× magnification, NA = 0.14, Mitutoyo) is used to collect the light on the achromatic metalens array, and another objective (10× magnification, NA = 0.28, Mitutoyo) is used to form the reimaging image from the achromatic metalens array on the camera (FLIR BFS-U3-200S6). All mounted components are then moved together along axial direction (*z*-direction) to capture the light image at different *x-y* planes in sequence. Finally, the light intensity profile from all captured images is stitched together to realize the intensity distribution along *z*-direction, as shown inSupplementary Fig. 3.

Using laser light sources, the focus spot images and focus efficiencies at 633 nm, 520 nm, and 455 nm are measured shown in Supplementary Fig. 6. The efficiencies are 39%, 55%, and 72% (efficiencies are normalized to the incident light) for 633 nm, 520 nm, and 455 nm superior to the previous reports on TiO_2_ metalens.

Because the dispersion at imaging plane is not standard linear. We implement spectrum calibration to rectify the nonlinearity between the displacement of a spectral component on the sensor plane and its wavelength. In practice, we perform spectrum calibration by imaging white LED lamp with filters at 5 different wavelengths. The Circle Hough Transform (CHT) is used to determine the optics center of each sub-lens for different wavelength. Supplementary Fig. 7 shows captured images for dispersion calibration.

**3.2 The spectrum calibration of metalens**

Once the spectrum distortion is calibrated, we then recover the true spectral radiance at each wavelength up to a constant scale factor. In practice, we perform spectrum calibration by imaging a white surface under white LED light and detecting the strong valley and peak at wavelengths 480 nm and 568 nm. Using this technique, we undistort the captured spectrum shown in Supplementary Fig. 8A to obtain the linear spectrum shown in Supplementary Fig. 8B, which closely matches the ground truth spectrum of the white LED lamp. This experiment demonstrates the ability of our system to recover high resolution spectrum.

The reflaction spectrum from a scene with a Color 24 Checker under the pre-mentioned white LED illumination are also use to check the spectrum response of the metalens through the whole visible wavelength range. As shown in Supplementary Fig. 9, our results show that all restructed spcetra for these 24 colors closely match the spectral power distributions of every patch, as measured by a spectrometer. For evaluation the performance of our calibration, conform that the metalens array can be used in the whole visible wavelength range.

**3.3 The spatial calibration of metalens**

For imaging and calibration, the spatial resolution of the metalens, the 1951 USAF resolution test chart, is used as a target object and a collimated beam was passed through the linear polarizer and a quarter-wave plate before being focused by an objective (7.5× magnification, NA = 0.21, Mitutoyo) to illuminate the target object. Optical setup for imaging resolution test chart is shown in Supplementary Fig. 10. The metalens is placed a focal length away from the object and paired with another object (7.5× magnification, NA = 0.21, Mitutoyo) to form an image on a CCD camera (FLIR BFS-U3-200S6). We adjusted the distance between the object and metalens based on the illumination wavelength. To reduce background signals, we used a polarizer paired with a quarter-waveplate in cross polarization.

The light field image formed by the transversely dispersive metalens array, in which each sub image of a single metalens unit contains the individual information of the target object. The reconstructed image is shown inSupplementary Fig. 11B after the rendering process. The smallest feature of objects that could be resolved was 3.10 μm in linewidth (group 7, element 3), which is consistent with the design target of the chromatic metalens array.

**3.4 The spatial and spectrum 4D imaging using metalens**

A white LED lamp is used as the broadband light source to illuminate the target object with incident angle of *q* = 16°. Circularly polarized light is generated by a linear polarizer and a quarter-wave plate. An objective (5× magnification, NA = 0.14, Mitutoyo) is used to collect the light to the achromatic metalens array, and another objective (10× magnification, NA = 0.28 Mitutoyo) is used to form the reimaging image from the achromatic metalens array on the camera (FLIR BFS-U3-200S6). The optical setup for the scene “META” character is shown in Supplementary Fig. 12. The position of the metalens array for the focused light field (FLF) camera follows the Gaussian lens formula: 1/*a* + 1/*b* = 1/*f*, where *f* is the focal length of a single metalens, *a* and *b* are the distance from the main lens image plane to the metalens array and the distance from the metalens array to the reimaging plane, respectively.

To demonstrate the capability of material discrimination using only the spectral information without depth hint. We captured a scene that have only one depth layer. Supplementary Fig. 13 shows a scene contain two characters at same depth with similar colors but have different spectrum response. Although both hands exhibit similar intensities in the RGB domain. We find that the spectral difference between (562nm) and (530nm) for “C” character is much bigger than the “V” character. Based on this feature, we employ a simple method that thresholds the quantity *r*(562) − *r*(530). Where *r*(*l*) denotes the spectral radiance at wavelength *l*. Supplementary Fig. 13D indicates the detected “C” character pixels in the captured frames.

**Supplementary Note 4: Light-field-spectrum reconstruction algorithm**

The imaging acquisition geometry of our spectral light field camera is shown in Supplementary Fig. 14. The transversely dispersive metalens array forms many dispersive-blurred sub-aperture images at the sensing plane. The placement of the transversely dispersive metalens array for focused spectral light-field imaging also follows the Gaussian lens formula:

|  | $\frac{\text{1}}{\text{a}}\text{+}\frac{\text{1}}{\text{b}}\text{=}\frac{\text{1}}{\text{f}_{\text{META}}}$ | (1) |
| --- | --- | --- |

Where $f_{\mathrm{META}}$, $a$ and $b$ represent the focal length of metalens, the distance for sample to metalens and the distance from metalens to sensor plane, respectively.

In Supplementary Fig. 14, we show the entire process of imaging a letter “A” (containing multiple wavelengths of light) through the SLIM system. The scene is first imaged through the main lens (part-1), then relayed by the metalens array for secondary imaging (part-2). Each metalens captures a part of the scene due to the difference in position (the images captured by adjacent metalens also have overlapping parts to calculate the depth information, part 3). Since the proposed metalens is designed to have transverse dispersion, each metalens captures a dispersive blurred imaging (the imaging between different wavelengths has small displacement in the lateral direction, so they are superimposed as if the photo is blurred), taking advantage of the additional information brought by this blurry, we could reconstruct the spectral information of each metalens image. After the spectral information reconstruction is completed, the clear spectrum images of each metalens is achieved. The depth information is obtained by calculating the parallax between adjacent metalens. At this point, both spectral and depth reconstruction can be achieved.

The 4D rendering pipeline for spectral light field imaging is given in Supplementary Fig. 15. Firstly, extracting sub-aperture dispersive-blurred image from captured raw image. Secondly, performed spectral reconstruction on the transversely dispersive-blurred image for every sub-aperture. After the spectral reconstruction process, the spectral data is put back to the original position. Thirdly, perform light field rendering on each sub image. The basic FLF camera rendering algorithm is shown schematically. First choose the pitch size and then select squares of that size from each sub-aperture image (marked in yellow). The reconstructed image is rendered by tiling the selected squares together. finally, rendering the color image according to the spectral information.

Our algorithm reconstructed the input dispersed gray image as multi-spectral cube, to obtain clear texture information without dispersive blurring. The reconstructed spectral image can be estimated from an input dispersive blurred image, by minimizing the following convex optimization:

|  | $\underset{\text{S}}{\text{argmin}} \left\Vert\text{Φ}\text{S-D} \right\Vert_{\text{2}}^{\text{2}}\text{+}\text{α}_{\text{1}}\left\Vert\text{∇}_{\text{xy}}\text{S} \right\Vert_{\text{1}}\text{+}\text{β}_{\text{1}}\left\Vert\text{∇}_{\text{λ}}\text{S} \right\Vert_{\text{1}}$ | (2) |
| --- | --- | --- |

The first term describes the data residual of our image formation model, $\left\| \text{·} \right\|_{\text{2}}$ is the L_2_ norm, used to constraint the data fidelity. $\boldsymbol{\Phi}$ describe the image degradation from multi-spectral data to dispersed gray image. $\mathbf{S}$, $\mathbf{D}$ are the spectral data and dispersed gray image. while the other terms are priors, $\text{α}_{\text{1}}$ and $\text{β}_{\text{1}}$ are the weights of corresponding terms respectively. $\left\| \text{·} \right\|_{\text{1}}$ is the L_1_ norm, where $\text{∇}_{\text{xy}}$ is a spatial gradient operator denote the difference of spectral data and image plane, $\text{∇}_{\text{λ}}$ is a spectral gradient operator denote the difference of spectral data at adjacent channel. The first prior is a traditional total variation term, ensuring sparsity of spatial gradients and removal of spatial artifacts. The second prior is a channel-wise total variation term, ensuring sparsity of spectral gradients and preserve the spectral consistence. To solve Eq. 2, we introduce auxiliary variables$\text{g}\left( \text{J}_{\text{1}} \right)$and $\text{h}\left( \text{J}_{\text{2}} \right)$ suggested in [1] to split our problem into three subproblems:

$$\text{f}\left( \text{S} \right)\text{=}\left\| \text{ΦS}\text{-}\text{D} \right\|_{\text{2}}^{\text{2}}\text{, g}\left( \text{J}_{\text{1}} \right)\text{=}\text{α}_{\text{1}}\left\| \text{J}_{\text{1}} \right\|_{\text{1}}\text{, h}\left( \text{J}_{\text{2}} \right)\text{=}\text{β}_{\text{1}}\left\| \text{J}_{\text{2}} \right\|_{\text{1}}\text{,}$$

so, the problem described as Eq. 2 is reformulated as:

|  | $\min_{\text{S}\text{,}\text{J}_{\text{1}}\text{,}\text{J}_{\text{2}}} \text{f}\left( \text{S} \right)\text{+g}\left( \text{J}_{\text{1}} \right)\text{+h}\left( \text{J}_{\text{2}} \right)$  $\text{subject to }\text{∇}_{\text{xy}}\text{S}\text{-}\text{J}_{\text{1}}\text{=0, }\text{∇}_{\text{λ}}\text{S}\text{-}\text{J}_{\text{2}}\text{=0.}$ | (3) |
| --- | --- | --- |

The ADMM solves this problem by optimizing Eq. 3 with respect to each variable iteratively. The scaled form of ADMM sub-problems is:

|  | $\text{S}^{\text{k+1}}\text{=}\min_{\text{S}} \text{f}\left( \text{S} \right)\text{+}\frac{\text{ρ}_{\text{1}}}{\text{2}}\left. \text{\vert}\text{∇}_{\text{xy}}\text{S}\text{-}\text{J}_{\text{1}}^{\left( \text{k} \right)}\text{+}\text{U}_{\text{1}}^{\left( \text{k} \right)} \right.\text{\vert}_{\text{2}}^{\text{2}}\text{+}\frac{\text{ρ}_{\text{2}}}{\text{2}}\left. \text{\vert}\text{∇}_{\text{λ}}\text{S}\text{-}\text{J}_{\text{2}}^{\left( \text{k} \right)}\text{+}\text{U}_{\text{2}}^{\left( \text{k} \right)} \right.\text{\vert}_{\text{2}}^{\text{2}}$  $\text{J}_{\text{1}}^{\text{k+1}}\text{=}\min_{\text{J}_{\text{1}}} \text{g}\left( \text{J}_{\text{1}} \right)\text{+}\frac{\text{ρ}_{\text{1}}}{\text{2}}\left. \text{\vert}\text{∇}_{\text{xy}}\text{S}^{\text{k+1}}\text{-}\text{J}_{\text{1}}^{\left( \text{k} \right)}\text{+}\text{U}_{\text{1}}^{\left( \text{k} \right)} \right.\text{\vert}_{\text{2}}^{\text{2}}$  $\text{J}_{\text{2}}^{\text{k+1}}\text{=}\min_{\text{J}_{\text{2}}} \text{h}\left( \text{J}_{\text{2}} \right)\text{+}\frac{\text{ρ}_{\text{2}}}{\text{2}}\left. \text{\vert}\text{∇}_{\text{λ}}\text{S}^{\text{k+1}}\text{-}\text{J}_{\text{2}}^{\left( \text{k} \right)}\text{+}\text{U}_{\text{2}}^{\left( \text{k} \right)} \right.\text{\vert}_{\text{2}}^{\text{2}}$  $\text{U}_{\text{1}}^{\text{k+1}}\text{=}\text{U}_{\text{1}}^{\text{k}}\text{+}{\text{∇}_{\text{xy}}\text{S}}^{\text{k+1}}\text{-}\text{J}_{\text{1}}^{\text{k+1}}$  $\text{U}_{\text{2}}^{\text{k+1}}\text{=}\text{U}_{\text{2}}^{\text{k}}\text{+}{\text{∇}_{\text{λ}}\text{S}}^{\text{k+1}}\text{-}\text{J}_{\text{2}}^{\text{k+1}}$ | (4-4) |
| --- | --- | --- |

Where $\text{U}_{\text{1}}$ and $\text{U}_{\text{2}}$ are Lagrange multipliers. The variable $\text{S}^{\text{k+1}}$ is updated by the gradient descent method. Where k and k+1represent the $k$-th and (k+1)-th iteration. The auxiliary variables $\text{J}_{\text{1}}$ and $\text{J}_{\text{2}}$ are updated by using a soft-thresholding operator, which is the proximal operator of L_1_ Norm given as:

|  | $\text{S}_{\text{θ}}\left( \text{x} \right)\text{=}\left\{ \begin{aligned} \text{ x-θ x>θ} \\ \text{ 0 }\left\vert\text{x} \right\vert\text{≤θ} \\ \text{ x+θ x<-θ} \end{aligned} \right.$ | (5) |
| --- | --- | --- |

where *θ* is a parameter that determines the step of the next update. The Lagrange multipliers $\text{U}_{\text{1}}$ and $\text{U}_{\text{2}}$ are then updated via gradient ascent to relate the objective function with constraints in Eq. 4. After we obtained the undispersed image, we use this information to reconstruct a hyperspectral image so that we can properly rely on the texture information. The reconstruction is performed by solving the equation below:

|  | $\underset{\text{S}}{\text{argmin}} \left\Vert\text{Φ}\text{S-D} \right\Vert_{\text{2}}^{\text{2}}\text{+}\text{β}_{\text{2}}\left\Vert\text{Φ}\text{∇}_{\text{xy}}\text{S}\text{-}\text{∇}_{\text{xy}}\text{D} \right\Vert_{\text{2}}\text{+}\text{γ}_{\text{1}}\left\Vert\text{M}_{\text{xy}}\text{⨀}\left( \text{S-P} \right) \right\Vert_{\text{2}}^{\text{2}}$ | (6) |
| --- | --- | --- |

The first term is still the data fidelity term, $\left\| \text{·} \right\|_{\text{2}}$ is the L_2_ norm, used to constraint the data fidelity. The second term represents the DoB (difference of blur) constraint, where $\text{Φ}$ describe the image degradation from multi-spectral data to dispersed gray image. $\text{∇}_{\text{xy}}$ is a spatial gradient operator denoted the difference of spectral data at image plane. $\text{S}$**,** $\text{D}$ are the spectral data and dispersed gray image. For the third term, M indicate the marginal mask of sub-images, $⨀$ denotes the pixel-wise production operation. $\text{S}$**,** $\text{P}$ are the spectral data and boundary information for sub-aperture image. During the light-field imaging, the scene is divided by each sub-aperture, margin spectral information at image boundary could be achieved precisely via this property. As a supplement to Fig. 2, Supplementary Fig. 22 shows a more complex scene to prove the robustness and reliability of our proposed spectral reconstruction algorithm.

**Supplementary Note 5: Trained spectrum super resolution algorithm**

To further improve the spectral resolution, we proposed a data driven spectrum super resolution algorithm using deep neural network. We use paired low-resolution spectral data and high-resolution spectral data as the input and output to train a spectral super resolution network. We trained the network on ICVL [2] dataset, the high-resolution spectral data is resized to 8 nm and 4 nm precision as paired data. The input of network is spectral data at 8 nm resolution, the output of network is spectral data at 4 nm resolution, and spatial information of the paired data is one-pixel to one-pixel correspondence. We propose 3D convolutional networks called Spectral Super-resolution Network (SSN), which are more suitable for spatial-spectral feature learning compared to 2D convolutional networks. The architecture of SSN is shown in Supplementary Fig. 16. In the input layer we are using 3×3×3 (channel×height×width) convolution kernel to learn both spatial and spectral structure. For the deeper layers, we take advantage of the inception network by using convolution kernels with four different sizes (1×1×1), (3×1×1), (5×1×1), (7×1×1) to learn spectral information at different scale.

We split the ICVL spectral dataset into training data and testing data, we trained our network on training data, and verify the effectiveness of SSN on the test data, the testing result of SSN is presented in Supplementary Fig. 17. Comparison of PSNR for the test spectral data by Bicubic and our method is displayed under the images. the proposed spectral super-resolution network (SSN) shows significant improvement, surpassing more than 2dB in PSNR over traditional method.

**Supplementary Note 6: Tradeoff between in-plane spatial resolution, spectral resolution, and depth resolution**

Before analyzing the tradeoff between in-plane spatial, spectral, and depth resolution, we wish to briefly define the resolution of these three variables. To make the discussion simple and clear, we use a dimensionless definition.

1. The in-plane spatial resolution is defined with pixel counts. For example, an image with $R_{\mathrm{spatial}}$pixels height and $R_{\mathrm{spatial}}$ pixels width, is defined with an in-plane spatial resolution of $R_{\mathrm{spatial}}$.
2. The spectral resolution is defined with spectral channel numbers $R_{\mathrm{spectral}}$. We assume that the dispersion is approximately uniform, which is consistent with our design. Therefore, a 50 channels spectral images for SLIM system (ranging from 450-650 nm) yields an actual spectral resolution of 4 nm.
3. The depth resolution is defined with depth layers $R_{\mathrm{depth}}$.For example, a 10-layer slices of scenes ranging from 20~30 cm yields an actual depth resolution of 1 cm.

**6.1 Tradeoff between the in-plane spatial resolution and the spectral resolution**

The spectral information is reconstructed from the dispersion blurred image, and the image formation process is shown in Supplementary Fig. 20. The light of different wavelengths from a single pixel is dispersed by metalens and transmitted to different positions on the imaging plane. The spectral information of different wavelengths of different pixels is superimposed (integrated) and captured by the sensor to form a dispersion blurred image. Therefore, when the size of the single-wavelength image is $\text{R}_{\text{spatial}}$ × $\text{R}_{\text{spatial}}$, the channel size is $\text{R}_{\text{spectral}}$, the dispersion blurred image captured by the camera will be$\text{R}_{\text{spatial}}$ × ($\text{R}_{\text{spatial}}$ + $\text{R}_{\text{spectral}}$), shown in Supplementary Fig. 20. The size of the reconstructed spectral data cube will be $\text{R}_{\text{spatial}}$ × $\text{R}_{\text{spatial}}$ × $\text{R}_{\text{spectral}}$.

Because the proposed metalens array is arranged in a tight form, the in-plane spatial resolution + spectral resolution of the image under a single metalens should be not greater than the lens diameter. The diameter of single metalens is denoted by $\text{D}_{\text{lens}}$, the diameter of single metalens at imaging plane is proportional to $\text{D}_{\text{lens}}$. The pixel size is denoted by *P*, and $\frac{\text{D}_{\text{lens}}}{\text{P}}$ is the space bandwidth product of single metalens, meaning the total information. Hence, the relationship between in-plane spatial resolution and spectral resolution is:

|  | $\text{R}_{\text{spectral}}\text{+}\text{R}_{\text{spatial}}\text{=ξ}\frac{\text{D}_{\text{lens}}}{\text{P}}$ | (7) |
| --- | --- | --- |

Here, $\text{ξ}$ is related with the size of the image of the metalens. ***This relationship shows that the summation of the in-plane spatial and spectral resolution is limited by the size of image captured by each metalens.***

**6.2 Tradeoff between depth resolution and spectral resolution**

According to the imaging principle of the focused light field camera, the depth resolution of a SLIM camera is related to the optional patch size of a single metalens. The final image is rendered by tiling the selected squares together. Choosing one pitch or another puts different world planes “in focus.” In other words, patches match each other perfectly only for one image plane behind the main lens. By the lens equation, this corresponds to a given depth in the real world. Thus, a different patch size would correspond to a different depth. The equivalent of refocusing is accomplished in the focused light field camera data through the choice of the patch size. Hence, the maximum depth resolution is half of the space bandwidth product $\frac{\text{D}_{\text{lens}}}{\text{P}}$ for the symmetry:

|  | $\text{R}_{\text{depth}}\text{=}\frac{\text{ξ}}{\text{2}}\frac{\text{D}_{\text{lens}}}{\text{P}}$ | (8) |
| --- | --- | --- |

In fact, the relationship between in-plane spatial resolution, spectral resolution and depth resolution is obtained by combining Eq. 7 andEq. 8 as follow:

|  | $\text{η(}\text{R}_{\text{spectral}}\text{+}\text{R}_{\text{spatial}}\text{)+}\text{2(1-η)R}_{\text{depth}}\text{=ξ}\frac{\text{D}_{\text{lens}}}{\text{P}}$ | (9) |
| --- | --- | --- |

Here, $\text{η}$ is an arbitrary coefficient lower than 1. Eq. 9 shows the limit of the summation of in-plane spatial resolution, spectral resolution, and depth resolution, which is related with space bandwidth product, as well as the tradeoff between them.

**6.3 Tradeoff between spectral resolution and numerical aperture**

In addition to the above-mentioned tradeoff relationship, by adjusting the position of metalens array, the object-image relationship is altered, and the image distance is extended. Therefore, we could obtain a larger dispersion width, thereby improving the spectral resolution of the imaging system, shown in Supplementary Fig. 21.

Here, the distance between the focal points of 450 nm and 650 nm in the focal plane is $w_{0}$, the dispersed spectrum width $w_{i}$ on imaging plane would be:

|  | $\text{w}_{\text{i}}\text{=}\frac{\text{b}}{\text{f}}\text{w}_{\text{0}}$ | (10) |
| --- | --- | --- |

In SLIM system, the spectral resolution is determined by the dispersed spectrum width on the sensor plane, which means the spectrum width varies over the image planes. The spectral resolution at imaging plane is:

|  | $\text{R}_{\text{spectral}}\text{=}\frac{\text{w}_{\text{i}}}{\text{P}}\text{=}\frac{\frac{\text{b}}{\text{f}}\text{w}_{\text{0}}}{\text{P}}$ | (11) |
| --- | --- | --- |

Due to the tight arrangement of metalens arrays, simply increasing the image distance in exchange for higher spectral resolution will cause imaging aliasing between adjacent metalens, shown in Supplementary Fig. 22.

Here, the imaging distance is *b*. To prevent the aliasing between adjacent metalens, the size of the image should not exceed the diameter of the lens, which requires the cone angle of the object ray bundles to meet the following conditions. The numerical aperture NA of an optical system such as an objective lens is defined by:

|  | $\text{NA=n}\sin\text{θ}$ | (12) |
| --- | --- | --- |

where $\text{n}$ is the refraction index of the surrounding medium. From Eq. 10 and Eq. 11, we derive the spectral resolution $\text{R}_{\text{spectral}}$ for SLIM with respect to the numerical aperture is:

|  | $\text{R}_{\text{spectral}}\text{=}\frac{\text{D}_{\text{lens}}\text{w}_{\text{0}}\text{n}}{\text{2f}\text{P*}\text{NA}}$ | (13) |
| --- | --- | --- |

By adjusting the numerical aperture of the imaging system and the position of the metalens array, to furthest extend the spectral resolution, the depth and in-plane spatial information should be compressed, as shown in the Supplementary Fig. 23. This evidently shows the tradeoff between the spectral resolution and spatial resolution.

***Increasing the resolution of any dimension will cause the loss of the resolution of the other two dimensions.*** Among them, due to the strong coupling of spectral information and in-plane spatial information, when the in-plane spatial resolution is improved, a subtle spectral resolution loss will occur, and vice versa. But for the depth resolution, the situation will be more complicated. Recording angular information requires a huge number of pixels. Even if we use the FLF (focused light-field camera) setting, coupling the spatial information and angle information together. Hence, when trying to increase the angular resolution, it will cause a large reduction of in-plane spatial and spectral resolution. The relationship between all 4D information is shown in Supplementary Fig. 24.

**6.4 Tradeoff between angular resolution and spectral resolution**

The angular samples for the focused light field camera for a given spatial point are obtained by different metalenses. Hence, the angular resolution $\text{R}_{\text{angular}}$ for the focused light field camera is determined by the optical geometry $a$ and $b$, the distance from spatial point to the metalens and the distances from metalens to imaging sensor.

Supplementary Fig. 24 shows the intermediate image space of a focused light field camera. The orange, green and blue lines are the chief rays associated with metalens *L*_1_, *L*_2_, and *L*_3_, respectively. Image points between plane $\text{v}_{\text{i}}$ and $\text{v}_{\text{i-1}}$ can be imaged by $i$ microlens, where plane $\text{v}_{\text{i}}$ and $\text{v}_{\text{i-1}}$ have a distance$b$[3]. in this case, the $R_{\mathrm{angular}}$ of the light field imaging system for point *A* is $i$. Supplementary Fig. 24 corresponds to the case $\text{i}\text{=2}$. The $R_{\mathrm{angular}}$ of focused light field camera could be defined as follow:

|  | $\text{R}_{\text{angular}}\text{=}\frac{\text{a}}{\text{b}}$ | (14) |
| --- | --- | --- |

Here, *a* represent the distance from spatial point to the metalens, *b* represent the distances from metalens to imaging sensor. The relationship between *a* and *b* could also be described with Gaussian lens formula, shown as follow:

|  | $\frac{\text{1}}{\text{a}}\text{+}\frac{\text{1}}{\text{b}}\text{=}\frac{\text{1}}{\text{f}_{\text{META}}}$ | (15) |
| --- | --- | --- |

Therefore, combine Eq. 14 and Eq. 15, the $\text{R}_{\text{angular}}$ could be represent as:

|  | $\text{R}_{\text{angular}}\text{=}\frac{\text{f}}{\text{b-f}}$ | (16) |
| --- | --- | --- |

From the analysis in the previous section, we show that the spectral resolution $\text{R}_{\text{spectral}}$ of the SLIM system is closely related to the imaging distance *b*. The relationship between $\text{R}_{\text{spectral}}$ and $\text{R}_{\text{angular}}$ could be derived as:

|  | $\text{R}_{\text{angular}}\text{=}\frac{\text{1}}{\frac{\text{P}\text{R}_{\text{spectral}}}{\text{w}_{\text{0}}}\text{-1}}$ | (17) |
| --- | --- | --- |

To prevent image aliasing between adjacent lenses, when adjusting the imaging distance, the NA of SLIM system must also be adjusted to match the imaging distance. With Eq. 13 and Eq. 17, the relationship between $\text{R}_{\text{angular}}$ and NA can be derived as:

|  | $\text{R}_{\text{angular}}\text{=}\frac{\text{1}}{\frac{\text{n}\text{ D}_{\text{lens}}}{\text{2f NA}}\text{-1}}$ | (18) |
| --- | --- | --- |

Eq. 18 show the tradeoff between $\text{R}_{\text{angular}}$, NA, and $\text{R}_{\text{spectral}}$ that the larger $\text{R}_{\text{spectral}}$ or the smaller NA will lead to smaller $\text{R}_{\text{angular}}$. We can find that the high requirement of spectral information will have cost in angular resolution.

**6.5 Bottleneck of the resolution tradeoffs**

**The fundamental limitation of SLIM system trade-off is the spatial bandwidth product.** Enlarge the sensor size allowing the entire system to receive more light, which raises the pixel numbers enabling the entire system of more detailed sampling. Therefore, the resolution of all four dimensions (*x + y + z + λ*) will be improved. Through the above analysis, to give a clear and simple conclusion, the tradeoff table is given in Supplementary Table. 3

**Reference**

1. Baek. SH *et al.*, *ACM Trans. Graph*. **36**(6), 1-2 (2017).
2. Arad. B *et al., Euro. Conf. Comput. Vision*, 19–34 (2016).

[3]. Zhu, S, et al., *Applied optics,* 57(1), A1-A11 (2018)

**Supplementary Video 1**：The video of SLIM system description.

**Supplementary Video 2**：The light field refocusing imaging of “M”, “E”, “T”, “A” in **Fig. 4** of the main text.


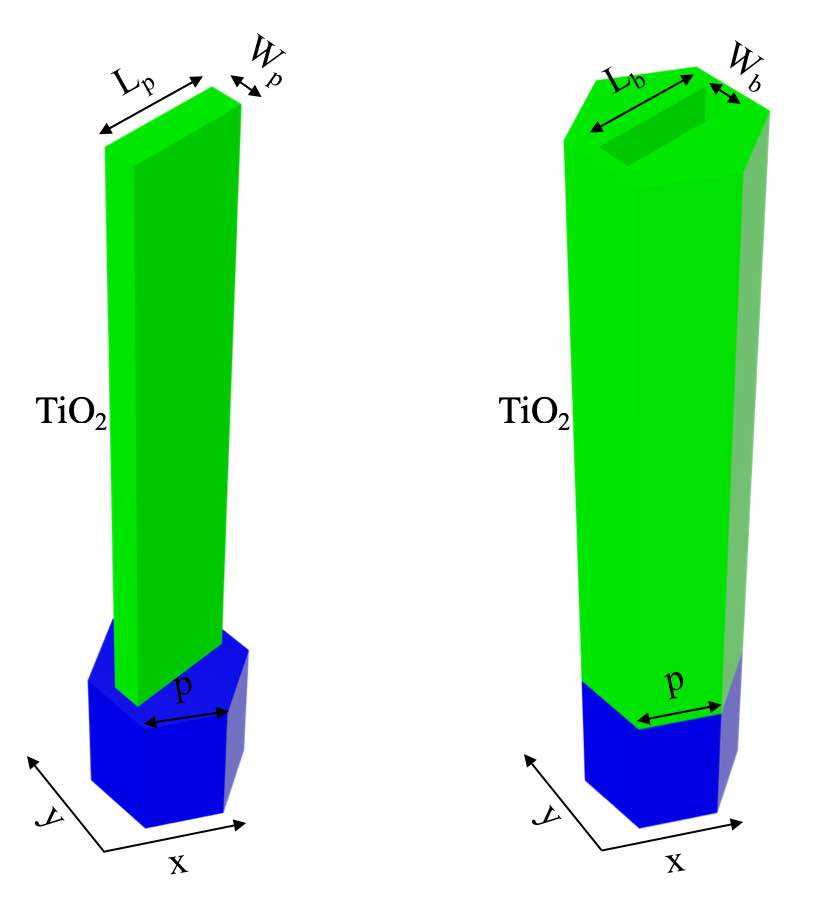


**Fig. S1:** **Schematics of the building blocks of TiO_2_-based metalens**. Two types of building blocks with a height of 800 nm are used: inverse and solid nanopillar structures. The lattice period *p* is 110 nm and the feature sizes, *W*_B_, *L*_B_, *W*_P,_ and *L*_P_, are optimized to satisfy the different phase requirements of transversely dispersive metalenses elaborated in Supplementary Table S1 and Table S2.

| Number | W_P_ (nm) | L_P_ (nm) | Compensation phase |
| --- | --- | --- | --- |
| 1 | 40 | 100 | 675 |
| 2 | 45 | 105 | 690 |
| 3 | 50 | 110 | 705 |
| 4 | 50 | 115 | 720 |
| 5 | 55 | 115 | 735 |
| 6 | 57 | 120 | 750 |
| 7 | 60 | 120 | 765 |
| 8 | 60 | 130 | 780 |
| 9 | 60 | 140 | 795 |
| 10 | 60 | 150 | 810 |
| 11 | 60 | 160 | 825 |
| 12 | 65 | 170 | 840 |
| 13 | 67 | 170 | 855 |
| 14 | 70 | 170 | 870 |
| 15 | 74 | 170 | 885 |
| 16 | 77 | 170 | 900 |
| 17 | 80 | 170 | 915 |
| 18 | 85 | 170 | 930 |
| 19 | 88 | 170 | 945 |

**Supplementary Table S1:** Feature sizes of TiO_2_ solid nanopillars.

| Number | W_B_ (nm) | L_B_ (nm) | Compensation phase |
| --- | --- | --- | --- |
| 1 | 100 | 170 | 960 |
| 2 | 95 | 170 | 975 |
| 3 | 90 | 170 | 990 |
| 4 | 85 | 170 | 1005 |
| 5 | 80 | 170 | 1020 |
| 6 | 75 | 170 | 1035 |
| 7 | 70 | 170 | 1050 |

**Supplementary Table S2:** Feature sizes of inverse TiO_2_ nanostructure.


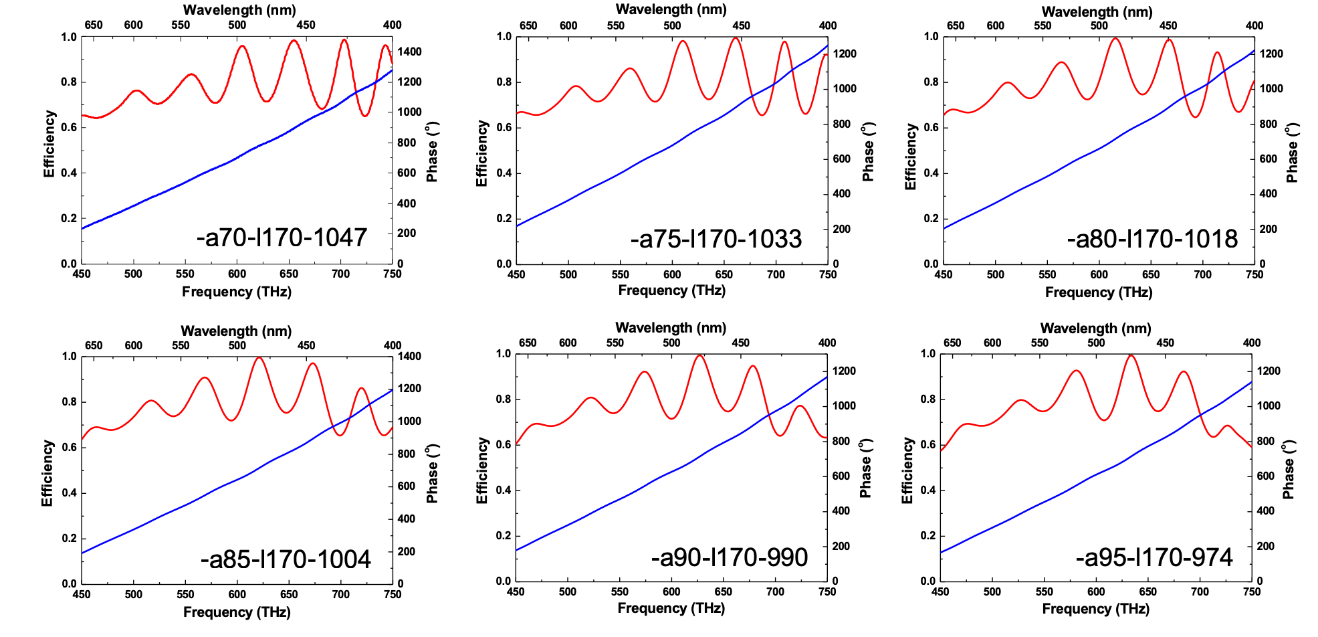

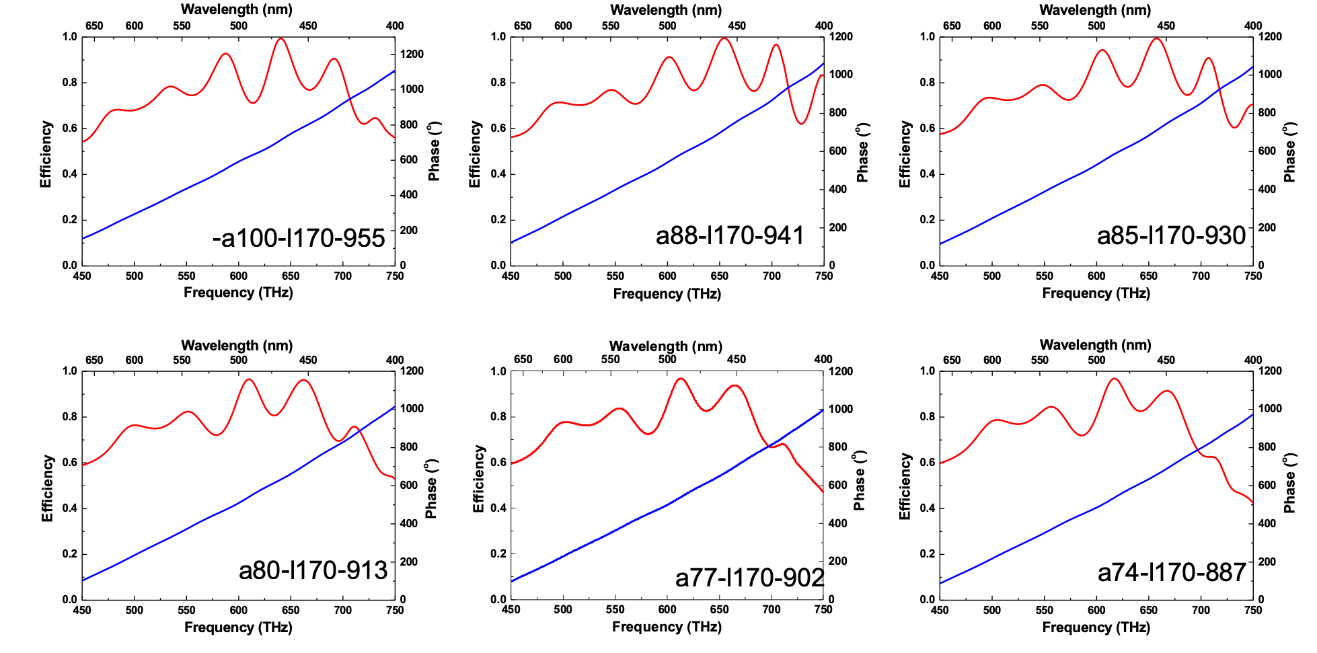


**
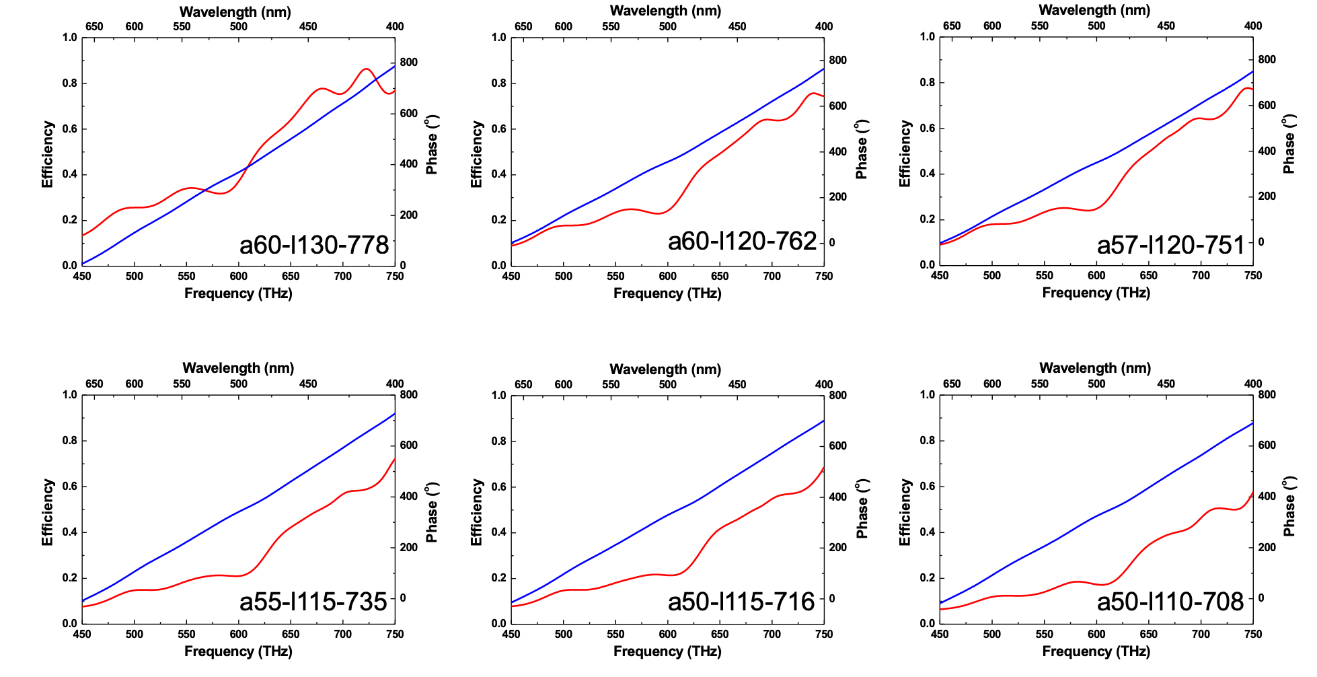
**

**
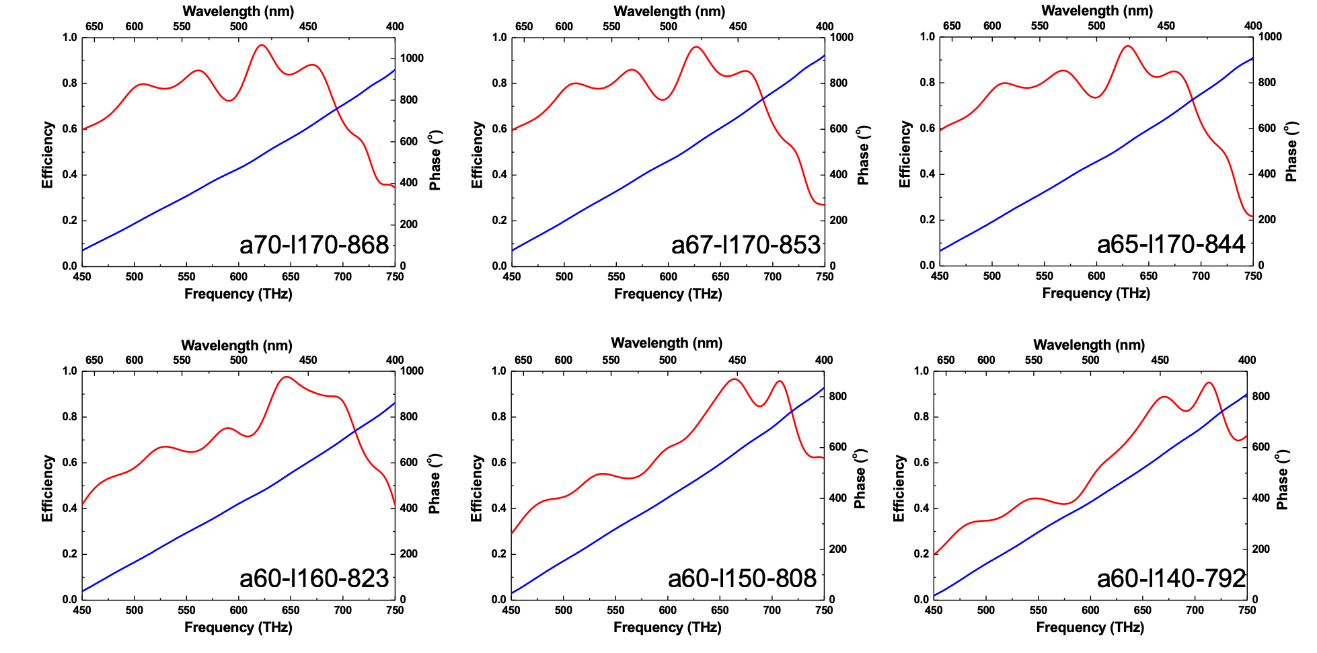

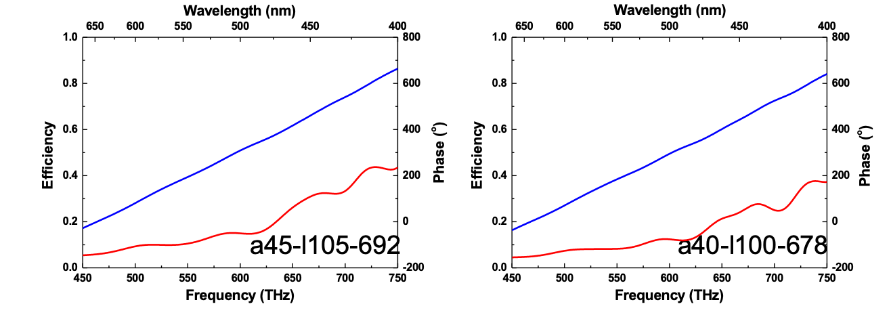
**

**Fig. S2: Transmission efficiency and phase shift of the unit elements**. Simulated transmission efficiency (red curves) and phase spectra (blue curves) of 26-unit elements for phase compensation from 675˚ to 1050˚, with phase interval being 375˚. The insets show the schematic for each structure.


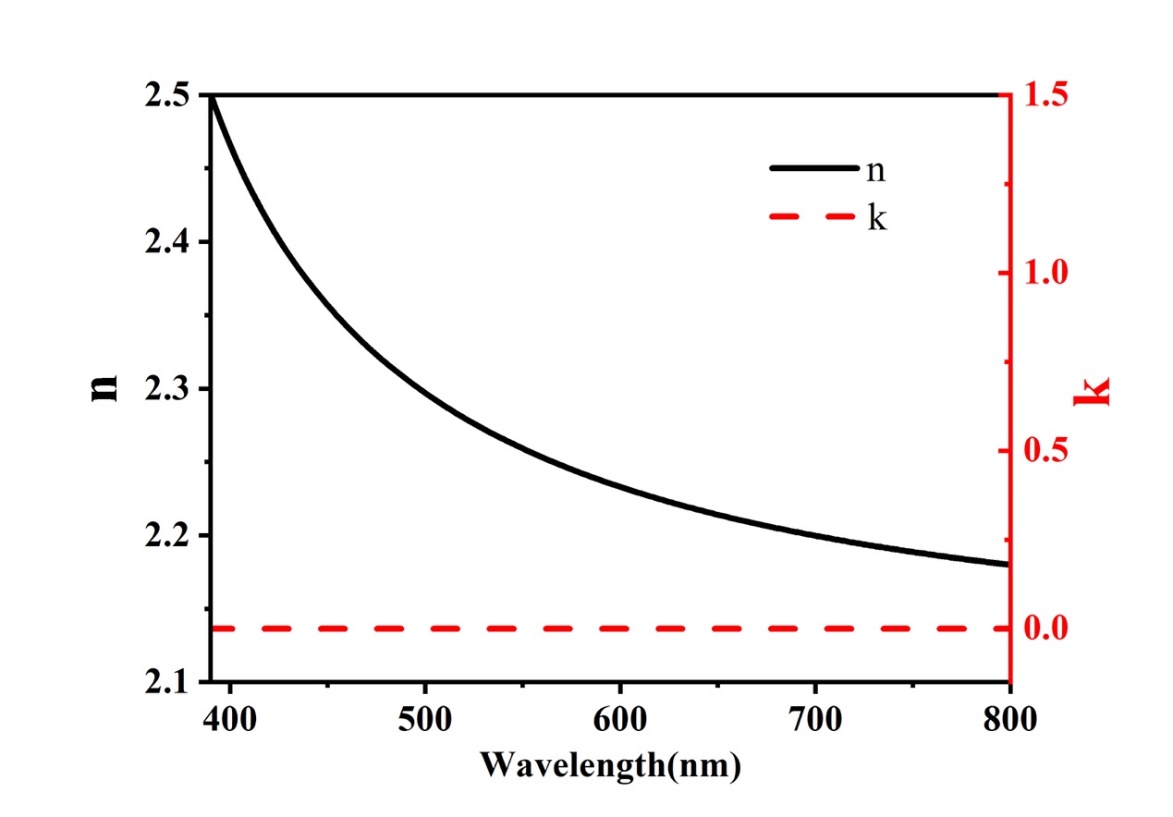


**Fig. S3: The refractive index and light extinction coefficient of TiO_2_ film.**


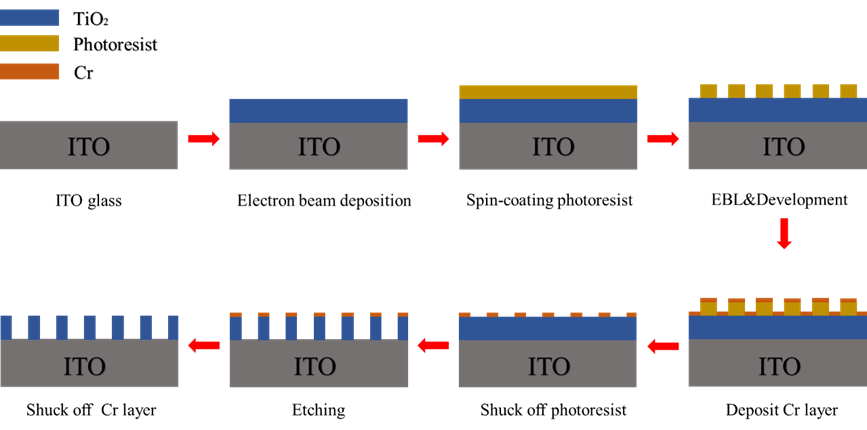


**Fig.. S4: The fabrication process for the TiO_2_ metalens.**


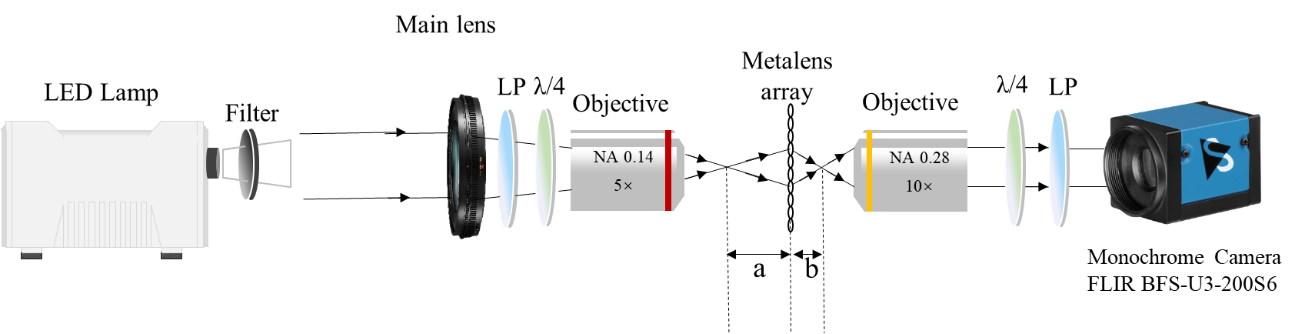


**Fig. S5: Schematic layout of spectral calibration for focused light field camera with transversely dispersive metalens array.** An optical bandpass filter is utilized to select the incident wavelength from the white LED lamp. Filter: optical bandpass filter, LP: linear polarizer, λ/4: quarter-wave plate, O: objective.


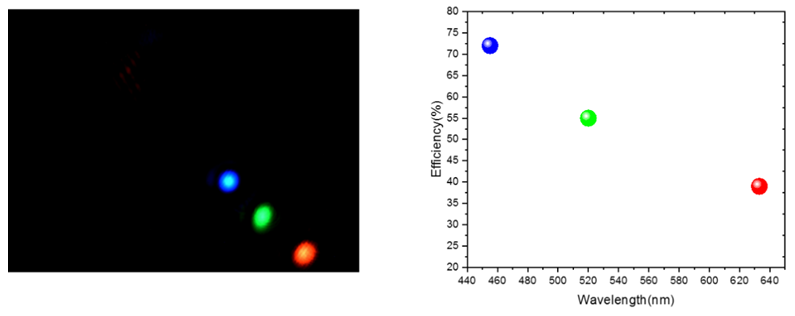


**Fig. S6: Measured focus spot images and efficiency at three wavelengths**. The focal spot and efficiency at wavelengths of 633 nm, 520 nm, and 455 nm.


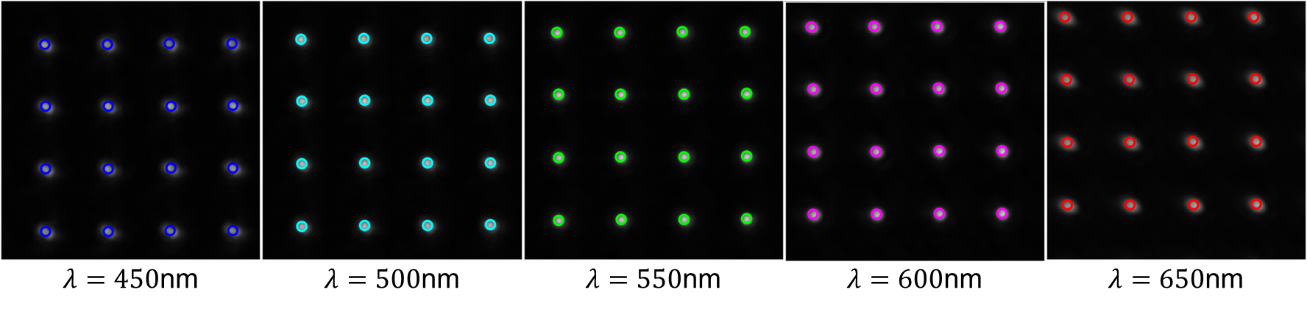


**Fig. S7: Optical center localization for different wavelength.** Bandpass filter with 10 nm FWHM (Thorlabs FB450-10. FB500-10, FB550-10, FB600-10, FB650-10) is used to generate monochromatic light to localize the optical center of each sub-lens at different wavelength.

**
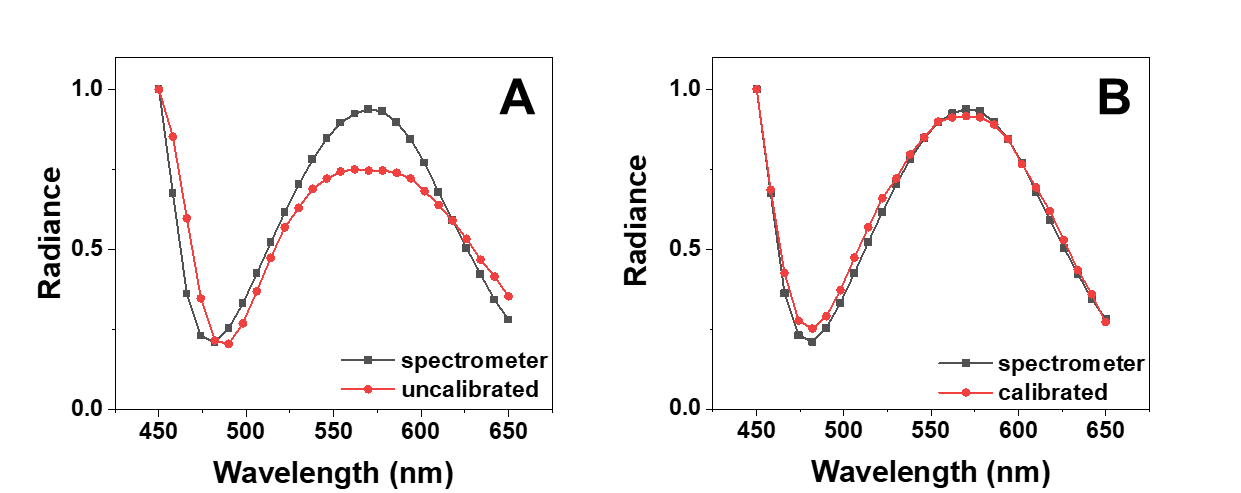
**

**Fig. S8: Spectrum radiance calibration. A**, spectrum response before calibration. **B**, spectrum response after calibration

**
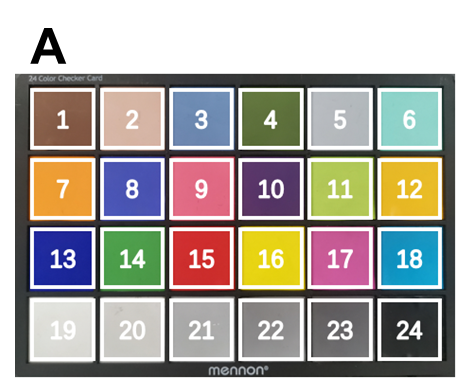
**

**
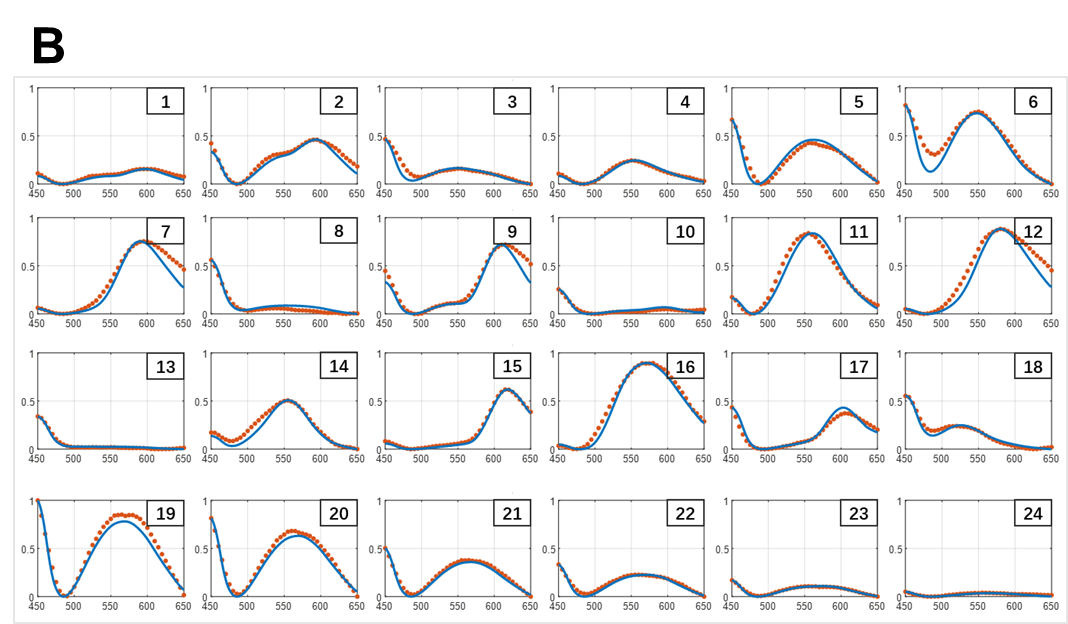
**

**Fig. S9:** **The working wavelength measurement.** **A**, color checker. **B**, Spectral plots of all 24 patches in the Color Checker.

**
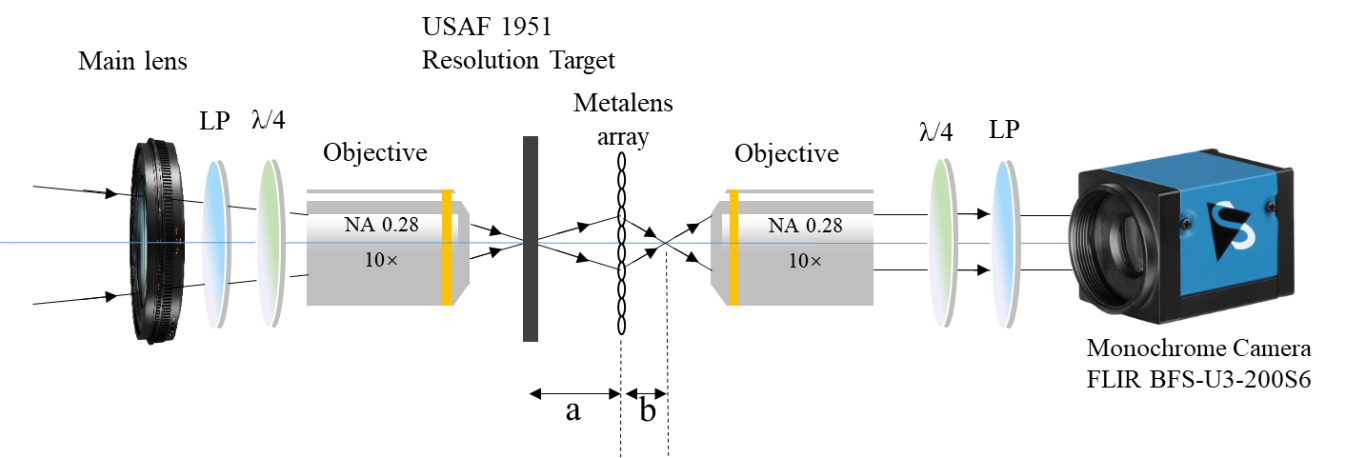
**

**Fig. S10: Optical setup for spatial resolution test.** To test the imaging resolution limits of the chromatic metalens array, the 1951 USAF resolution test chart, is used as a target object. The resolution test chart is illuminated by a white LED incoherent light source with a 450nm filter (FHWM 10 nm) collimated by a 10× object lens to eliminate aberration. The image of metalens array is relayed by a 50× object lens.

**
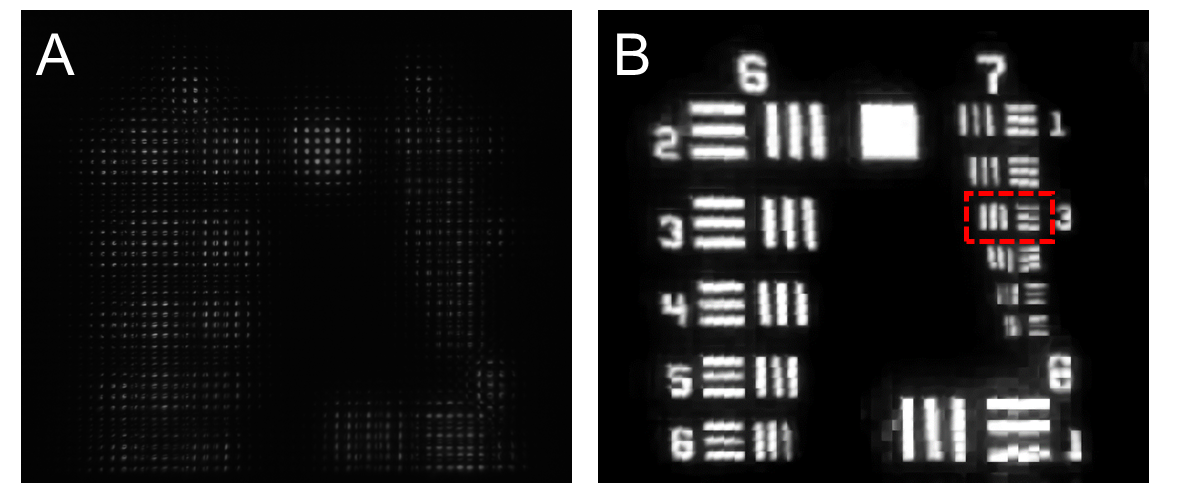
**

**Fig. S11: Schematic layout of focused light field camera with achromatic metalens array for resolution chart.** **A**, camera raw data. **B**, reconstructed all-focus image.

**
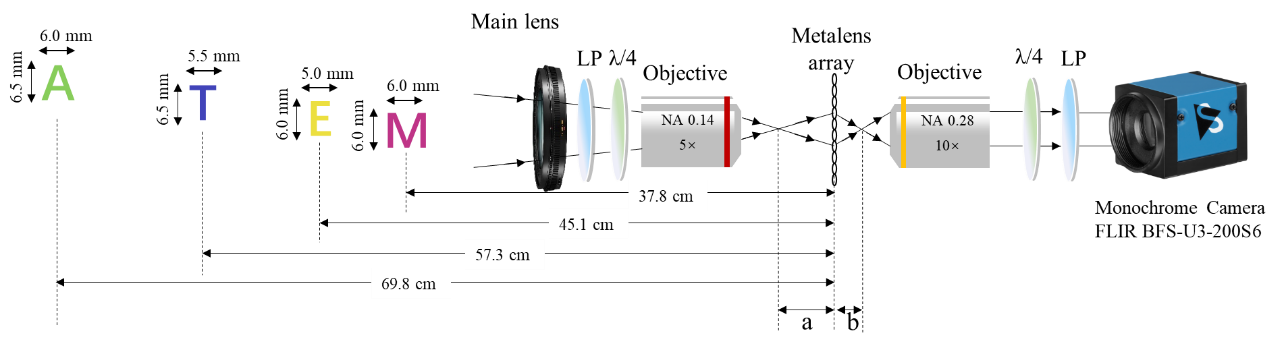
**

**Fig. S12: Schematic layout of focused light field camera with transversely dispersive metalens array for the scene “META” character.** Filter: optical bandpass filter, LP: linear polarizer, λ/4: quarter-wave plate, O: objective. The separated letters “META” are mounted on a linear stage (ODL220/M, Thorlabs) with different distance from the metalens.


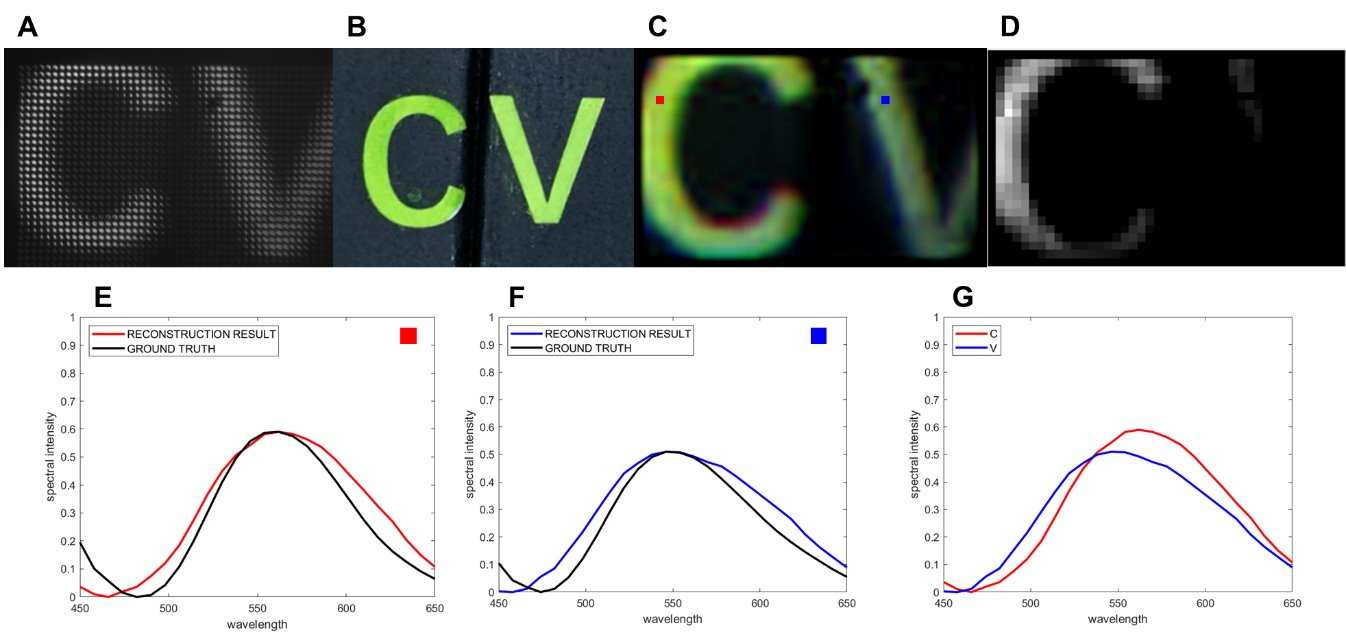


**Fig. S13: Scenes captured by the focused light field camera with transversely dispersive metalens array for similar colors with different spectrum response. A**, camera raw data. **B**, samples. **C**, color reconstruction. **D**, material discrimination. **E**, spectral compare for red spot position, red line represents SLIM reconstructed spectral curve, black line represents spectrometer result. **F**, spectral compare for blue spot position, blue line represents SLIM reconstructed spectral curve, black line represents spectrometer result. **G**, spectral compare for red spot and blue spot.

**
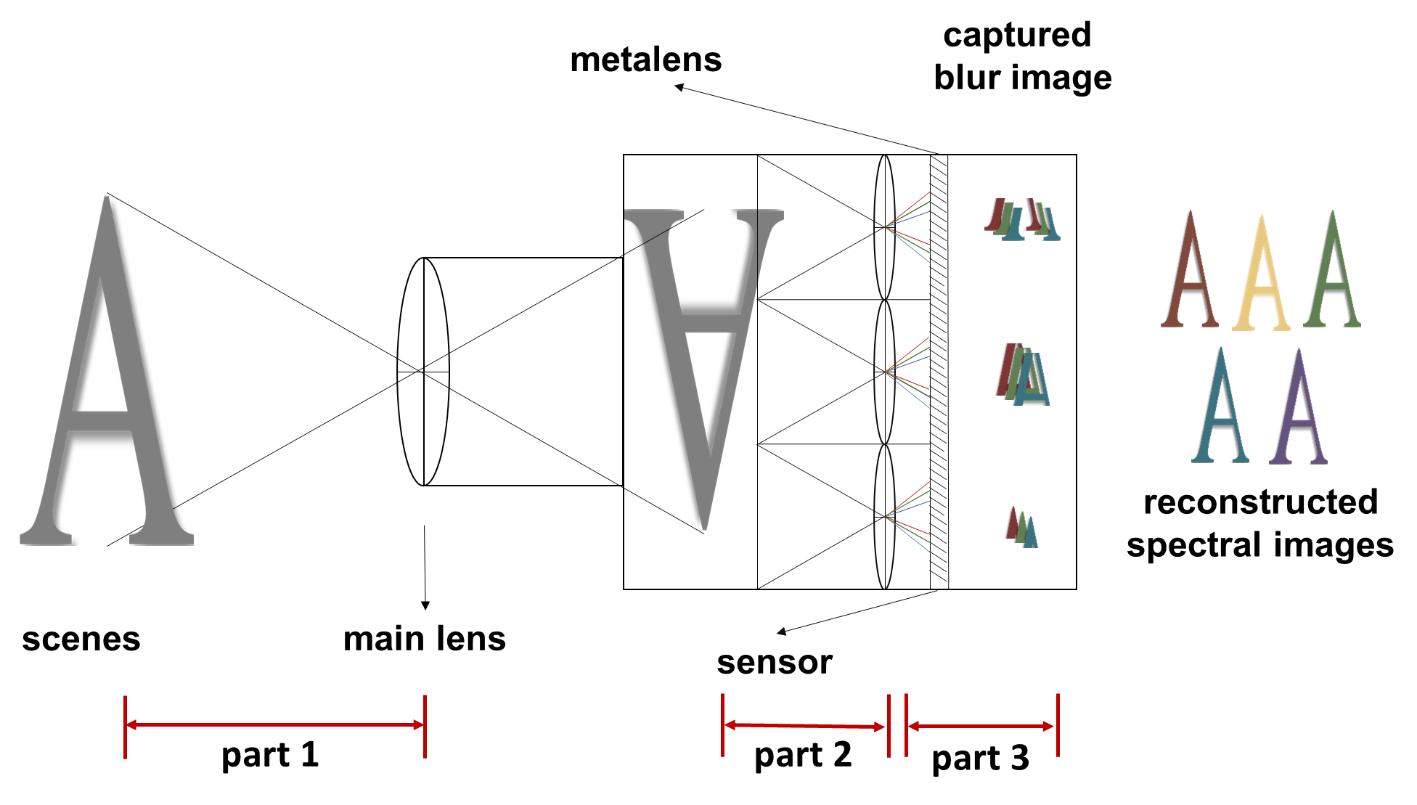
**

**Fig. S14: Detailed illustration of the SLIM image formation.** The focused light field imaging acquisition geometry when proper focusing is achieved. The transversely dispersive metalens array forms many sub-images at the sensor plane.


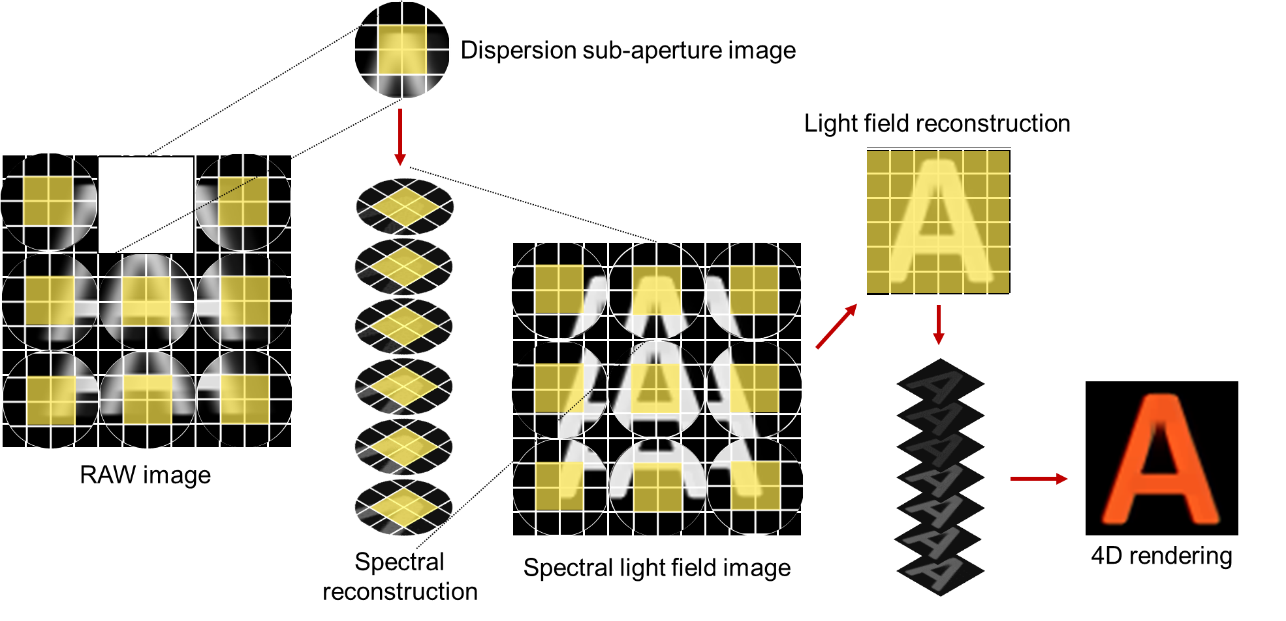


**Fig.. S15: 4D rendering pipeline**


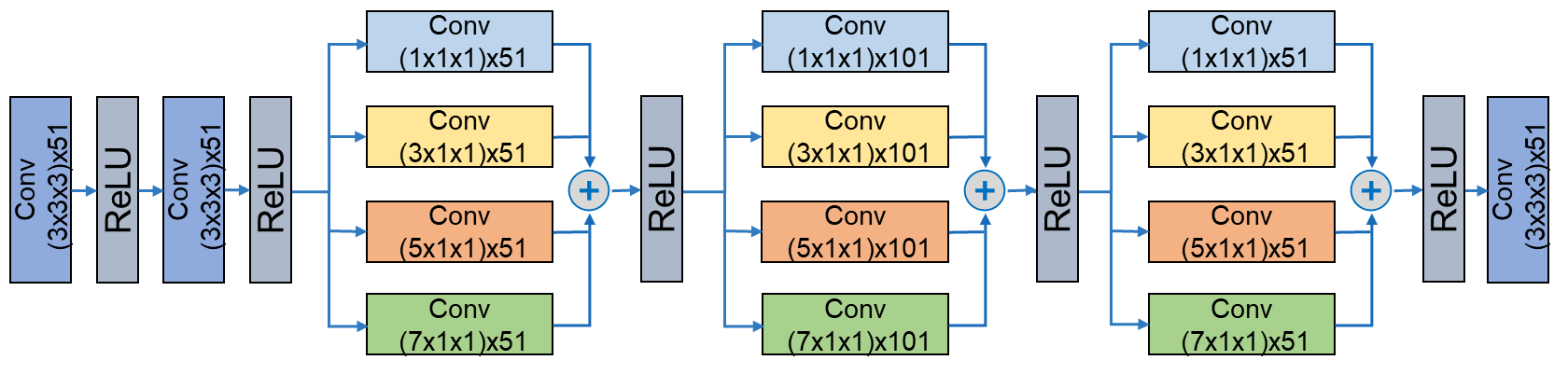


**Fig. S16: The architecture of our proposed spectral super resolution network**


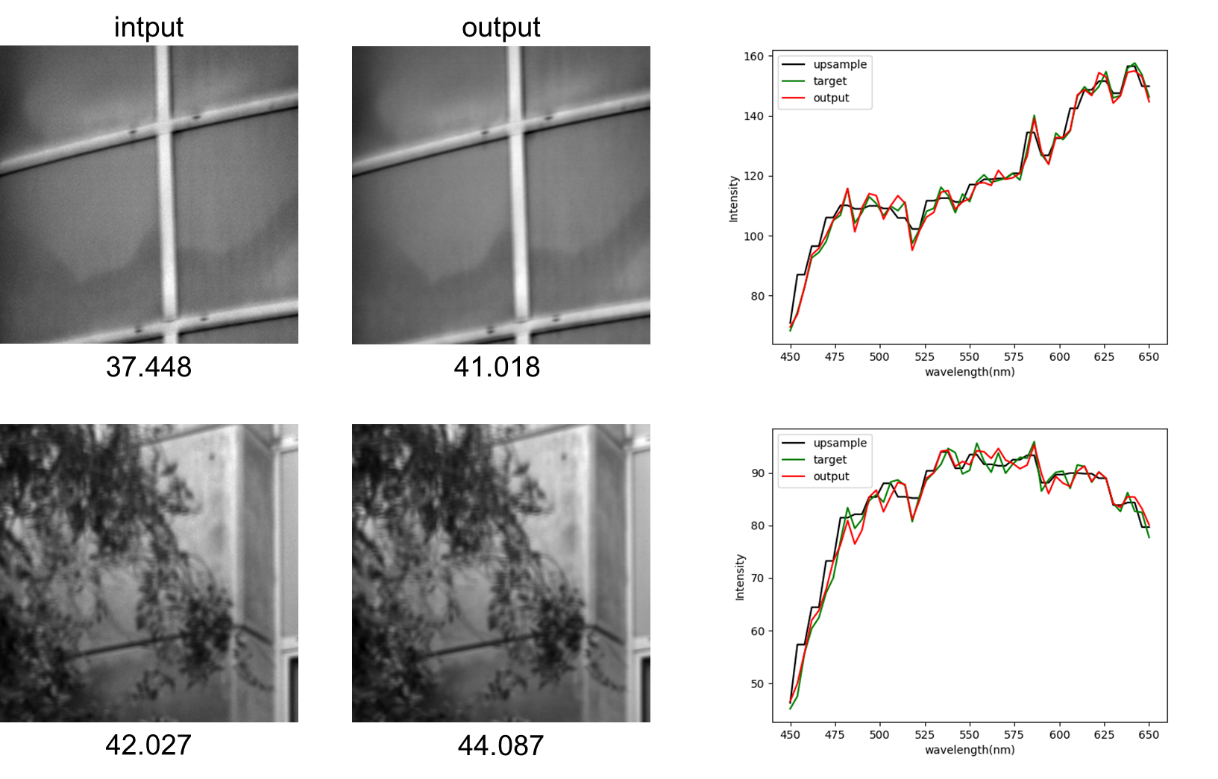


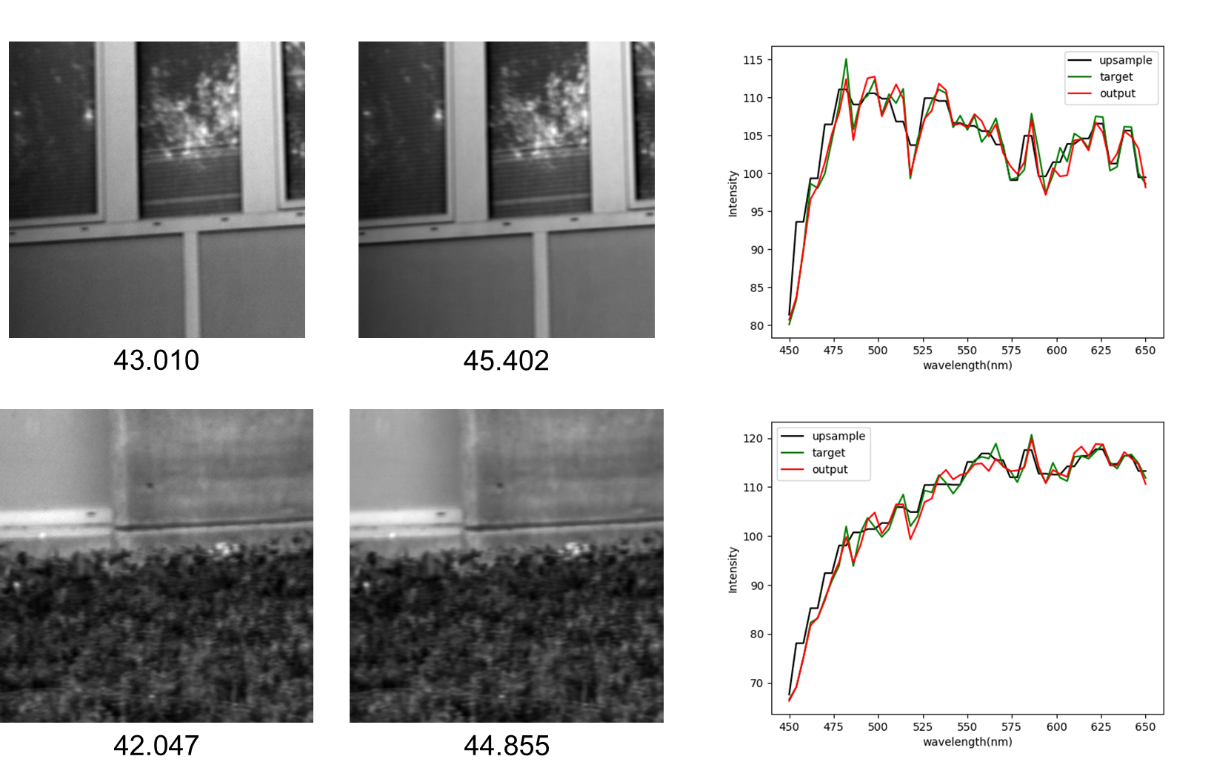


**Fig. S17: Visual comparisons of the super-resolution results.** The first column is the gray image of spectral data resized to 8 nm precision. The second column is the output of our Spectral Super-resolution Network (SSN) of spectral precision of 4 nm. The third column is spectral plot of randomly chosen points, the plot of bicubic method is draw in black, the plot of proposed method is draw in red line, the plot of ground truth is draw in green line.


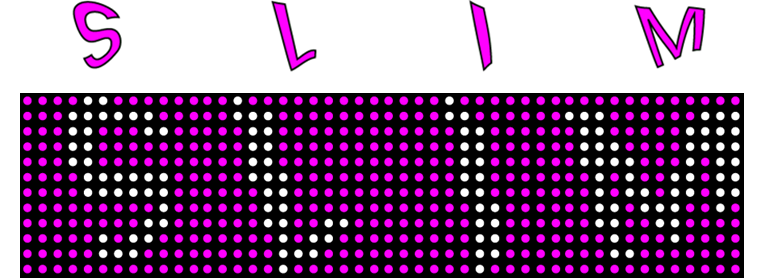


**Fig. S18: Pattern design for experiment of Fig.. 5.**


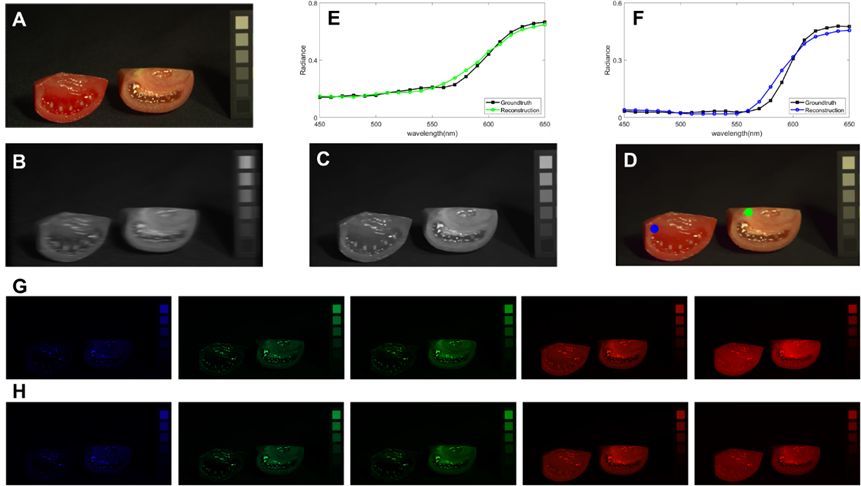


**Fig. S19: Numerical simulation results of spectral reconstruction algorithm for SLIM.** A. The original RGB image of spectral data. B. The simulated dispersion image using the forward model same as SLIM. C. The reconstructed gray image. D. The reconstructed color image synthesis from reconstructed spectral data. E. The spectral plot of green position, the green line is reconstructed result, the black line is ground truth. F. The spectral plot of blue position, the blue line is the reconstructed result, the black line is the ground truth. G. The original single wavelength images are presented. H. The reconstructed single wavelength images are presented.

**
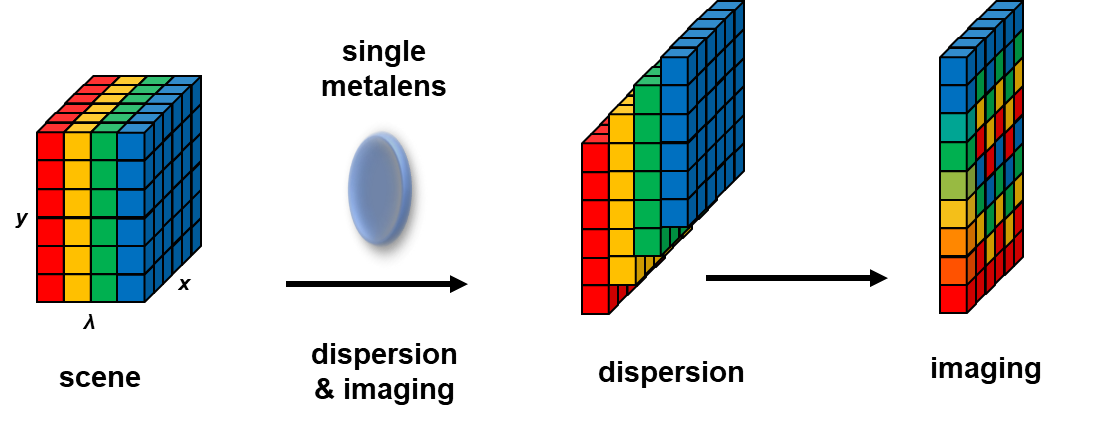
**

**Fig. S20: Dispersion image formation with transversely dispersive metalens.**


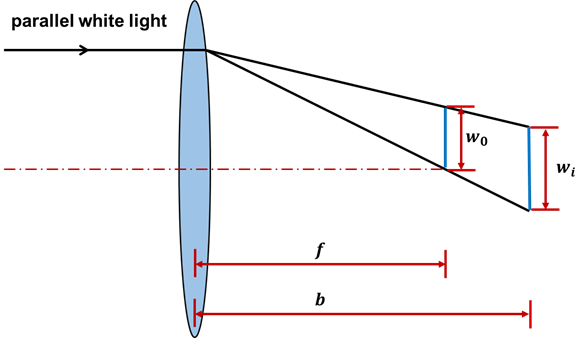


**Fig. S21: Extended spectral resolution by changing the imaging distance.** Where $\boldsymbol{f}$ is the focal length and $\boldsymbol{b}$ is the imaging distance. $\boldsymbol{w}_{\mathbf{0}}$ represents the dispersed spectrum width at the focal plane, and $\boldsymbol{w}_{\boldsymbol{i}}$ represents the dispersed spectrum width at the imaging plane.

**
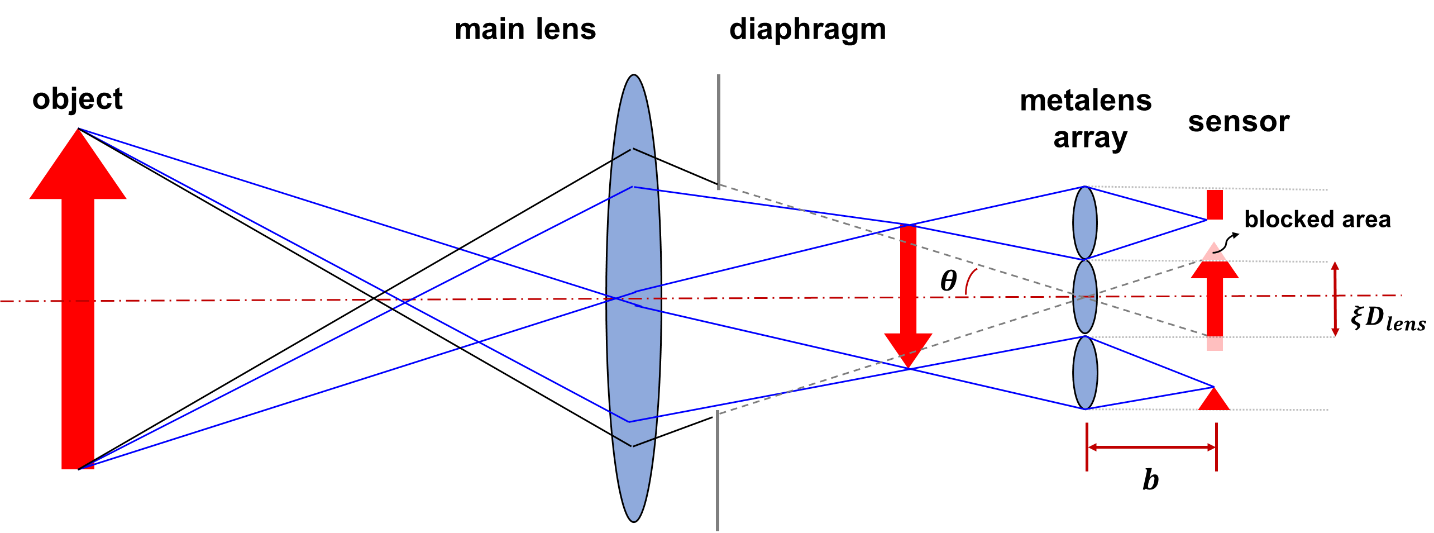
**

**Fig. S22: Image formation of SLIM. The object light forming the aliased image is indicated by the dotted line and the aliased areas are represented by transparent images. Object light causing aliasing is blocked by the diaphragm.**


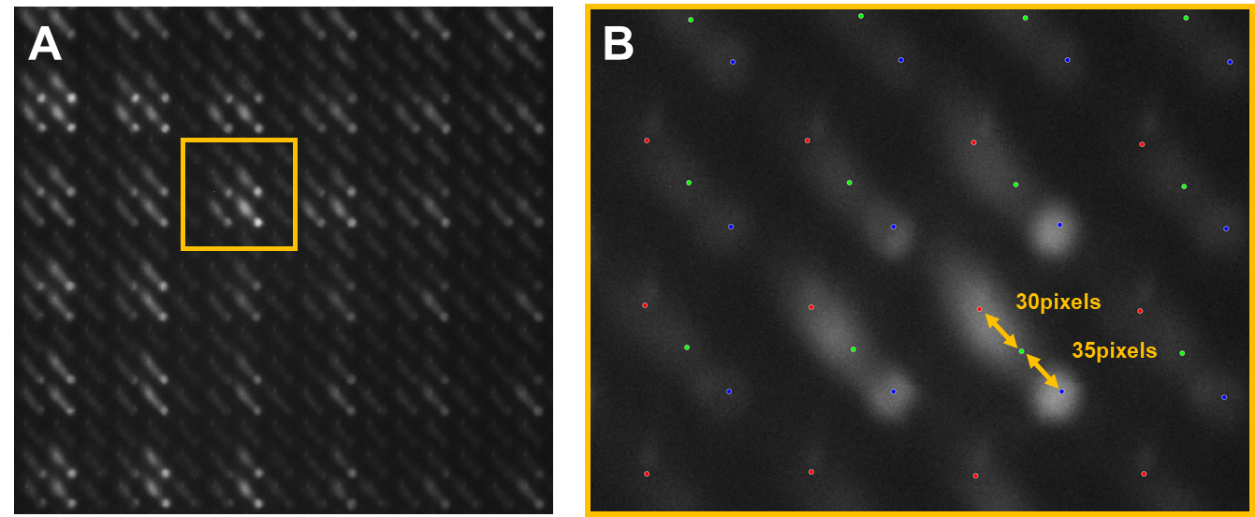


**Fig. S23: Enlarged dispersion width.** A. Raw data of **Fig.. 5**; B. Position of 450 nm, 550 nm and 650 nm spot are marked in blue, green, and red, respectively.


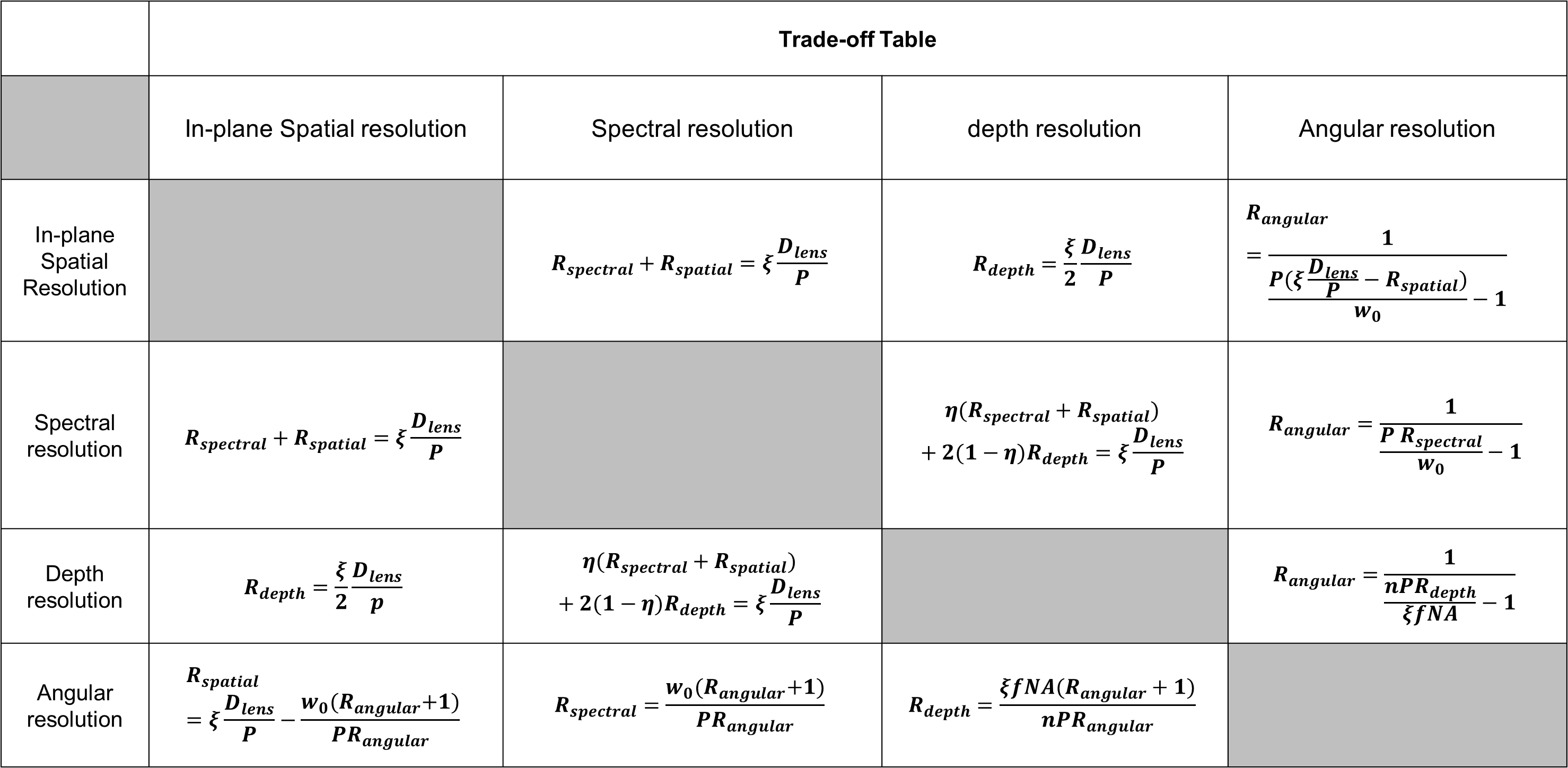


**Table. S3: Tradeoff table between in-plane spatial resolution, spectral resolution, depth resolution and numerical aperture.**

**
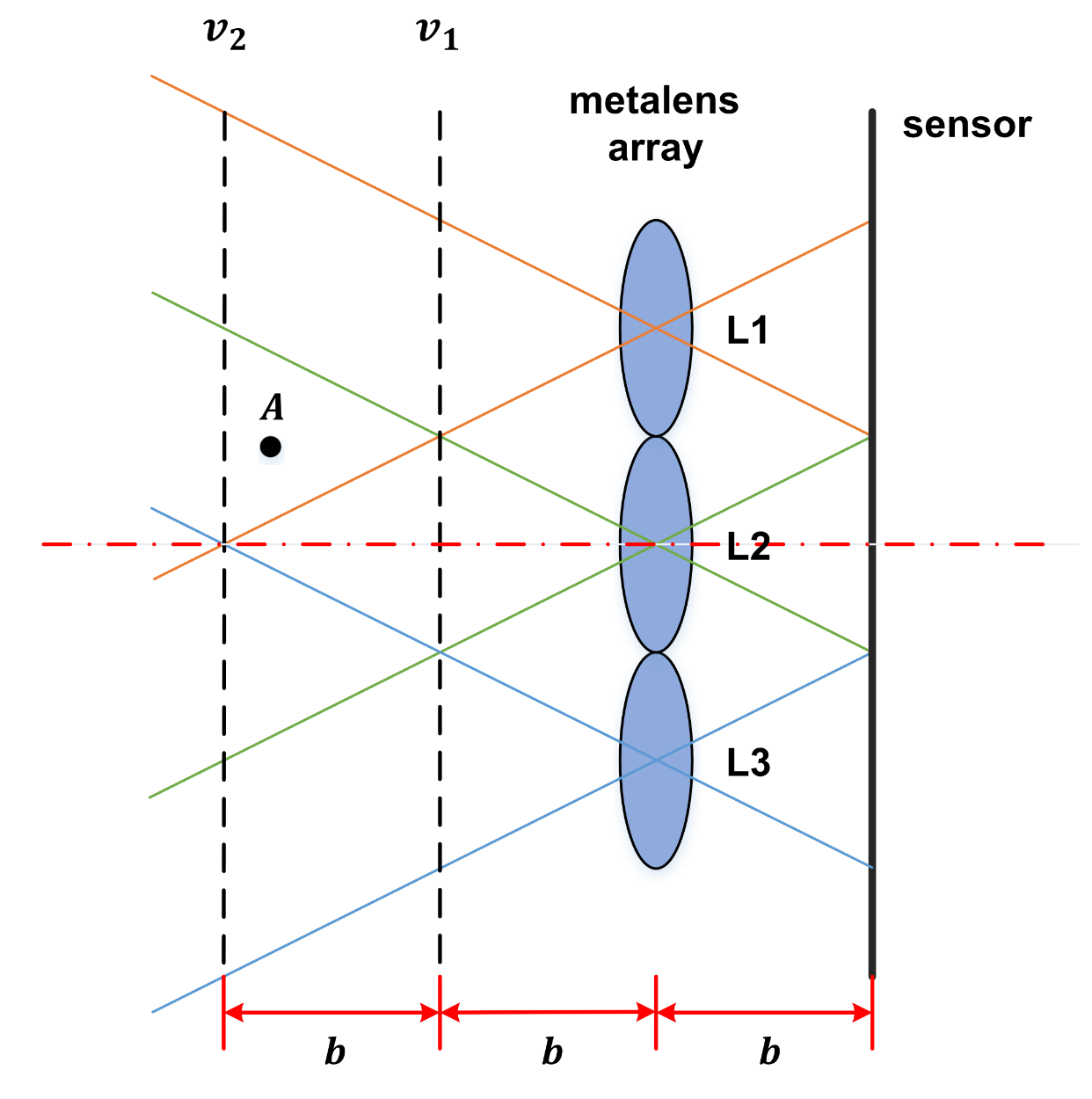
**

**Fig. S24: intermediate image space of focused light field camera**
