# Supplementary material for: Ultra-compact snapshot spectral light-field imaging
Source: Nat Commun. 2022 May 18;13:2732. doi: 10.1038/s41467-022-30439-9 (PMC9117667; doi:10.1038/s41467-022-30439-9)
Supplement: Supplementary file 3 — Description of Additional Supplementary Files [file 41467_2022_30439_MOESM3_ESM.docx]

**Description of Additional Supplementary Files:**

**Supplementary Movie 1:** System diagram of Ultracompact snapshot spectral light-field imaging

**Supplementary Movie 2:** Refocusing of figure4
